# Differences in Overweight and Obesity Prevalence among Young Men from Twelve Middle Eastern and Asian Countries Living in Saudi Arabia

**DOI:** 10.3390/healthcare10040690

**Published:** 2022-04-06

**Authors:** Reham I. Alagal, Nora A. AlFaris, Jozaa Z. AlTamimi, Naseem M. Alshwaiyat, Aryati Ahmad, Riyadh A. Alzaheb, Nora M. AlKehayez

**Affiliations:** 1 Department of Physical Sports Sciences, College of Education, Princess Nourah Bint Abdulrahman University, Riyadh 11671, Saudi Arabia; rialagal@pnu.edu.sa (R.I.A.); naalfaris@pnu.edu.sa (N.A.A.); jzaltamimi@pnu.edu.sa (J.Z.A.); 2 School of Nutrition and Dietetics, Faculty of Health Sciences, Universiti Sultan Zainal Abidin, Kuala Nerus 21300, Terengganu, Malaysia; sh_naseem@yahoo.com (N.M.A.); aryatiahmad@unisza.edu.my (A.A.); 3 Nuffield Department of Primary Care Health Sciences, University of Oxford, Oxford OX2 6GG, UK; 4 Department of Clinical Nutrition, Faculty of Applied Medical Sciences, University of Tabuk, Tabuk 71491, Saudi Arabia; ralzaheb@ut.edu.sa

**Keywords:** overweight, obesity, young men, Middle East, Asia

## Abstract

Objectives: This study was conducted to assess differences in the prevalence of overweight and obesity among young men from twelve Middle Eastern and Asian countries who live in Riyadh, Saudi Arabia. Methods: This study used a cross-sectional design and was conducted in Riyadh, Saudi Arabia. The body weight and height of 3600 young men (aged 20 to 35 years) were measured using standardized methods. The sociodemographic characteristics of the participants were collected using face-to-face interviews. Results: Overweight and obesity was reported in 48.3% of the study sample; 42.2% were overweight, and 6.2% were obese. The overweight and obesity rate was associated with the nationality of the participants. The lowest rate of overweight and obesity was observed among participants from Bangladesh (19.4%), while the highest rate was reported among participants from Egypt (67.5%). Older age and longer residency duration were associated with overweight and obesity risk among the subjects. Conclusion: The outcomes revealed a fairly high prevalence of overweight and obesity among participants. The notable disparity in overweight and obesity rates among participants of different nationalities is confirmed. The risk of overweight and obesity among young men in Saudi Arabia is associated with nationality, age, and residency duration.

## 1. Introduction

The prevalence of overweight and obesity continues to rise at an alarming rate globally [1,2]. Obesity is a severe health problem that is associated with an increased risk of many diseases, such as diabetes, cardiovascular disease, iron deficiency anemia, and many types of cancer [3,4,5]. Adulthood overweight and obesity is a multifactorial health problem that is influenced by the interaction of several risk factors, including biological and ecological factors [6,7]. The fast rise in the rate of overweight and obesity is mainly linked to ecological influences [8]. Modern life is strongly associated with dramatic changes in people’s lifestyles, especially among young adults. These changes are mostly characterized by unhealthy dietary patterns, such as high junk food consumption, a low consumption of vegetables and fruits, and sedentary behaviors [9]. This highlights the importance of adopting effective strategies, such as nutrition education and lifestyle modification, to fight overweight and obesity [10].

Young adults are more vulnerable to fast weight gain than any other age group [11]. This weight gain often starts during adolescence and continues to worsen during young adulthood [12]. Weight gain among young adults can be linked to dramatic lifestyle transitions experienced during this stage of life [13]. Common features of this transitional lifestyle include a high consumption of fast foods and high-sugar beverages, low physical activity levels, and frequent screen time, such as television watching, video gaming, and smartphone use [14,15,16]. Overweight and obese young adults commonly suffer from psychosocial distress and mental health problems, such as depression and anxiety [17]. In addition, they may have a greater susceptibility to obesity-related diseases, such as cardiovascular disease and diabetes, which may continue and worsen during middle age and the older stages of adulthood [18]. The management of obesity and its comorbidities in young adults is crucial for life-long optimal health, but it is challenging. This could be due to young adults having minimal symptoms, being less likely to receive healthcare services, and having difficulties complying with medical therapy [10]. Therefore, managing overweight and obesity during young adulthood requires healthcare providers to be aware of young adults’ age-specific needs, and to use proper strategies to handle them [19,20].

In the past few decades, Saudi Arabia has undergone a rapid socioeconomic evolution that is associated with an elevation in the occurrence of numerous chronic diseases in the country [21]. One major aspect of these changes is the continuous rise in overweight and obesity rates among individuals living in Saudi Arabia, including both sexes and different age groups [22]. The elevated consumption of fast foods in the usual diet, at the expense of fruits and vegetables, and the decline in physical activity levels, are blamed for this rise [23,24,25]. Moreover, Saudi Arabia is one of the top oil producers at the global level and an economically emerging country in the Middle East region. This makes Saudi Arabia an attractive destination for workers from different countries. Immigrants form approximately half of the gross labor power and most employees in the private sector in Saudi Arabia [26]. The fifth Saudi census revealed that an estimated 8.5 million non-citizen inhabitants form about 30% of the population. Additionally, the foreign population in Saudi Arabia is predominantly male, making up about 70% of non-Saudi residents [27]. This is particularly interesting because having migrants from different countries offers the opportunity to conduct comparative studies to detect disparities in disease incidence and associated factors among people from different countries. Assessing differences in the prevalence of overweight and obesity and associated risk factors allows researchers and decision-makers to identify significant characteristics targeted in obesity prevention initiatives in population subgroups with high prevalence rates. Currently, the available literature does not provide any data about overweight and obesity rates and associated factors in men from various countries who live in Saudi Arabia. The outcomes of the current study will be valuable to the healthcare system in Saudi Arabia, which is invited to adopt priority initiatives to reduce overweight and obesity incidence in different subgroups of the population. These initiatives should be tailored to satisfy the demands of various population subgroups. Thus, this study aimed to assess differences in the prevalence of overweight and obesity among young men from twelve Middle Eastern and Asian countries who live in Saudi Arabia.

## 2. Methods

### 2.1. Study Design and Participants

The current study is part of a research project titled “Relationship between Obesity, physical Activity and Dietary pattern among men living in the Kingdom of Saudi Arabia” (ROAD-KSA). The study design is cross-sectional. The present study was conducted from February to June of 2019 in Riyadh, the capital city of Saudi Arabia. The study participants were recruited from public places in Riyadh City, such as public parks and shopping malls, using stratified cluster sampling based on geographical locations in Riyadh City. The inclusion criteria were men aged 20–35 years, living in Riyadh, free of any physical impairment, and being from one of seven Middle Eastern countries (Saudi Arabia, Egypt, Yemen, Syria, Jordan, Sudan, and Turkey) and five Asian countries (Pakistan, Afghanistan, India, Bangladesh, and the Philippines). In addition to the host country, the choice of other countries included in this study was based on the number of citizens (at least 250,000) of these countries who live in the Kingdom of Saudi Arabia. Informed consent was signed by the participants in accordance with the Helsinki Declaration. The study protocol was approved by the research ethics committee of Princess Nourah bint Abdulrahman University, Riyadh, Saudi Arabia.

### 2.2. Sociodemographic Data

Sociodemographic data were collected from the participants by trained dietitians during face-to-face interviews. The data included the following sociodemographic characteristics: nationality, age, duration of residency in Saudi Arabia, household type, marital status, educational level, and monthly income.

### 2.3. Anthropometry Data

The body weight and height of the participants were measured by trained dietitians using standardized methods. The weight of participants was measured to the nearest 0.1 kg by a calibrated digital scale while wearing minimal clothing and no shoes. Their height was measured to the nearest 0.1 cm by a calibrated portable stadiometer while in the full standing position without shoes. Body mass index (BMI) was calculated by dividing weight (kg) by squared height (m^2^). Participants were grouped according to BMI into the categories of underweight (<18.5), normal weight (18.5–24.9), overweight (25–29.9), and obese (≥30) [28].

### 2.4. Statistical Analysis

IBM SPSS Statistics for Windows (version 26. Armonk, New York, United States, 2019) was used for the data analysis. For all of the variables of this study, there were no missing data. Categorical variables were analyzed by the Chi-square test and are presented as frequencies and percentages. A one-way ANOVA was used to analyze continuous variables, and the results are reported as means and standard deviations. To evaluate whether there were significant differences across subgroups, the Tukey post hoc test was performed. To find the characteristics linked to overweight and obesity risk, univariate and multivariate logistic regression analyses were conducted. Multivariate logistic regression analysis was used after adjusting for the following sociodemographic variables: nationality, age, residency duration, household type, marital status, educational level, and monthly income. Two-tailed tests were used to generate all of the stated *p* values. When the *p* value was less than 0.05, the differences were deemed statistically significant.

## 3. Results

**Figure 1:**
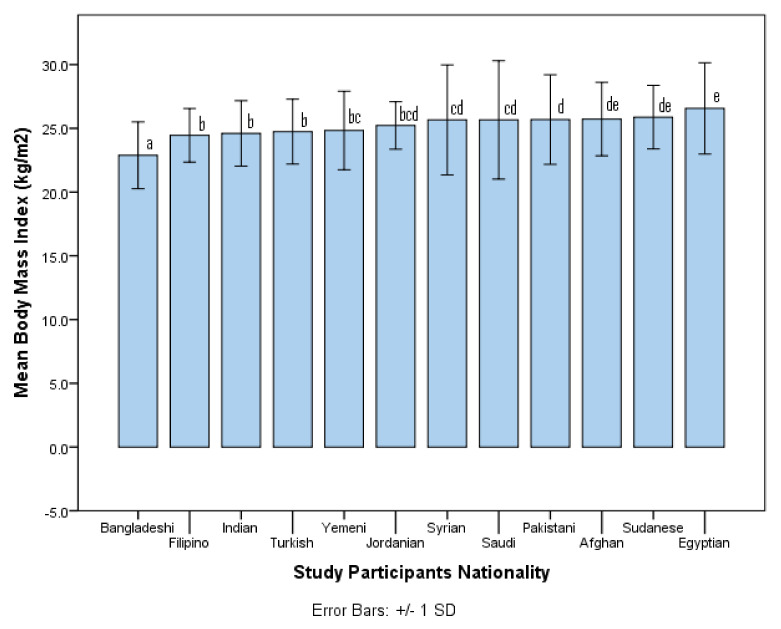
Bar chart illustrating mean body mass index for study participants from different countries. Bars labeled with different letters represent significantly different means (*p* < 0.05) based on the Tukey post hoc test.

In total, 3600 young men participated in the current study. The sociodemographic characteristics of the participants are presented in Table 1. By nationality, the lowest number of participants were from Turkey (*n* = 203; 5.6%), and the highest number of participants were from the Philippines (*n* = 379; 10.5%). The mean age of the subjects was 29.6 (SD = 3.2) years and their mean period of residency in Saudi Arabia was 7.2 (SD = 7.0) years. Only 18.9% of participants lived in households with their families, whereas the remaining participants lived away from their families. About half of the participants (53.3%) were single, whereas the remaining subjects were married. The educational level of 36.6% of participants was a college degree or higher, whereas the rest of the participants had a secondary school or lower level of education. The monthly income of 26.9% of participants was high (≥USD 1000), whereas it was low (˂USD 1000) for the remaining participants. Lastly, the mean BMI of the subjects was 25.1 (SD = 3.2). By nationality, the lowest and highest mean BMIs were found among subjects from Bangladesh (22.9; SD = 2.6) and Egypt (26.6; SD = 3.6), respectively (see Figure 1). There were statistically significant differences (*p* < 0.001) in mean BMI among subjects from different countries.

The prevalence of overweight and obesity among participants, stratified by nationality, is presented in Table 2. Overweight and obesity was observed among 48.3% of the subjects. By nationality, the lowest and highest rates of overweight and obesity were observed among subjects from Bangladesh (19.4%) and Egypt (67.5%), respectively. A relatively high overweight and obesity prevalence was also seen among participants from Sudan (67.0%), Afghanistan (56.8%), Jordan (55.7%), Syria (51.5%), Saudi Arabia (50.9%), and Pakistan (48.4%). Moreover, overweight and obesity prevalence among Yemeni, Turkish, Indian, and Filipino participants ranged from 41.4% to 44.1%. Obesity was observed in 6.2% of all subjects. By nationality, the lowest rate of obesity was observed among participants from Bangladesh (0.9%), while the highest rate was reported among participants from Pakistan (13.7%). Moreover, obesity prevalence was relatively high among participants from Saudi Arabia (13.5%), Egypt (12.5%), Syria (10.2%), and Afghanistan (7.6%). On the other hand, a relatively low rate of obesity was observed among subjects from the Philippines (1.1%), Jordan (1.8%), Sudan (2.5%), Turkey (3.0%), India (3.4%), and Yemen (5.1%). The prevalence of overweight and obesity was statistically significant (*p* value < 0.001) across participants of various nationalities. Table 3 shows the matrix of post hoc association coefficients for different pairs of participant subgroups based on their nationality.

Table 4 shows the risk of overweight and obesity among participants based on sociodemographic factors. Compared with Bangladeshi participants, subjects of other nationalities had a significantly higher risk of overweight and obesity (the adjusted odds ratio (OR) ranged from 2.43 (Turkish subjects) to 7.59 (Sudanese subjects), *p* = 0.001). Furthermore, overweight and obesity was found to be significantly associated with increasing age (adjusted OR = 1.07, *p* = 0.001) and residency period in Saudi Arabia (adjusted OR = 1.05, *p* = 0.001). Nevertheless, the outcomes of the multivariate analysis were not significant for the remaining sociodemographic characteristics: household type (adjusted OR = 0.87, *p* = 0.262), marital status (adjusted OR = 1.04, *p* = 0.689), educational level (adjusted OR = 1.22, *p* = 0.061), and monthly income (adjusted OR = 1.21, *p* = 0.067).

## 4. Discussion

The current study provides a comprehensive overview of the rates of overweight and obesity among young men from twelve countries who live in Saudi Arabia. The results revealed that overweight and obesity was observed among around half of the subjects. Obesity is considered a major public health problem in Saudi Arabia, with high prevalence rates at the global level [21,29]. A nationwide study examining obesity and its associated factors in Saudi Arabia revealed that 28.7% of Saudi individuals aged 15 years and older were obese (females: 33.5%; males: 24.1%) [22]. In another study, the national obesity rate among Saudi adults aged 18 years and over was 21.7% (females: 25.5%; males: 17.9%). Furthermore, 14.8% of young adults (20–29 years) were obese [30].

The current study’s findings demonstrate notable disparities in overweight and obesity rates among participants of different nationalities. These differences could be related to specific lifestyle determinants, such as usual dietary patterns, eating cultural foods, and levels of physical activity [31,32,33,34,35,36]. For example, the diet of Saudis tends toward a high consumption of refined and high-protein foods, and low consumption of plant-based foods [37]. In contrast, Indians consume high amounts of grains such as rice, and low amounts of high-protein foods [38]. In addition, there are variations in physical activity levels among young men from different countries [35,36]. Most young men in Bangladesh have relatively moderate or high physical activity levels [39]. For example, they use bicycles frequently for transportation. In contrast, young Saudi men have relatively low physical activity levels and high levels of screen time [25].

Assessing risk factors related to overweight and obesity provides an opportunity to find relevant variables that may be applied in strategies to reduce overweight and obesity in groups with high prevalence rates [40]. In this study, several sociodemographic characteristics were associated with the risk of overweight or obesity. The nationality of participants was one of these characteristics. This could be due to disparities in lifestyle patterns driven by cultural values and ethnic norms, such as diet and average physical activity levels [33,34,35,36,41]. The age of participants was another characteristic associated with the risk of overweight and obesity. This could be explained by the tendency of adults to lead more inactive lifestyles and perform fewer physical activities as they age [42]. A longer residency duration in Saudi Arabia was associated with the risk of overweight or obesity. This could be explained by the country’s urbanization and growth, and the impact of these changes on individuals’ lifestyle decisions [25]. The health of immigrants is likely to diminish as they spend more time in a new society. Cultural factors, social and economic changes, and changes in food and physical activity patterns that are associated with migration may cause health problems [43,44].

This study has some limitations. First, the study design (cross-sectional) cannot determine cause–effect relationships between overweight and obesity and the investigated sociodemographic characteristics. Second, this study did not include young women and adults from other nationalities living in Saudi Arabia that are less represented in the country. Third, only young men living in Riyadh City were recruited. Nevertheless, the present study still offers valuable results regarding differences in overweight and obesity prevalence among young men from twelve countries who live in Saudi Arabia.

## 5. Conclusions

The current study found a fairly high prevalence of overweight and obesity among participants. The findings demonstrate notable disparities in overweight and obesity rates among participants of different nationalities. The risk of overweight and obesity among young men in Saudi Arabia is associated with nationality, age, and the duration of their residency in the country.

## Figures and Tables

**Table 1:** Sociodemographic variables and body mass index of study subjects (*n* = 3600).

Variables	Frequencies/Means	%/SD
Participants’ Nationality		
Saudi	289	8.0%
Egyptian	289	8.0%
Yemeni	335	9.3%
Syrian	293	8.1%
Jordanian	280	7.8%
Sudanese	276	7.7%
Turkish	203	5.6%
Pakistani	306	8.5%
Afghan	303	8.4%
Indian	297	8.3%
Bangladeshi	350	9.7%
Filipino	379	10.5%
Age (years)	29.6	3.2
Residency Duration (years)	7.2	7.0
Household Type		
Non-family household	2920	81.1%
Family household	680	18.9%
Marital Status		
Single	1919	53.3%
Married	1681	46.7%
Educational Level		
Low (secondary school or less)	2284	63.4%
High (college degree or more)	1316	36.6%
Monthly Income		
Low (˂USD 1000)	2630	73.1%
High (≥USD 1000)	970	26.9%
Body Mass Index (kg/m^2^)	25.1	3.2

**Table 2:** Overweight and obesity prevalence among study participants stratified by their nationality.

Study Participants	*n* (%)	Body Weight Status *			
		Underweight	Normal	Overweight	Obesity
All	3600 (100%)	31 (0.9%)	1829 (50.8%)	1518 (42.2%)	222 (6.2%)
Saudi	289 (8.0%)	7 (2.4%)	135 (46.7%)	108 (37.4%)	39 (13.5%)
Egyptian	289 (8.0%)	0 (0.0%)	94 (32.5%)	159 (55.0%)	36 (12.5%)
Yemeni	335 (9.3%)	0 (0.0%)	194 (57.9%)	124 (37.0%)	17 (5.1%)
Syrian	293 (8.1%)	4 (1.4%)	138 (47.1%)	121 (41.3%)	30 (10.2%)
Jordanian	280 (7.8%)	0 (0.0%)	124 (44.3%)	151 (53.9%)	5 (1.8%)
Sudanese	276 (7.7%)	1 (0.4%)	90 (32.6%)	178 (64.5%)	7 (2.5%)
Turkish	203 (5.6%)	0 (0.0%)	116 (57.1%)	81 (39.9%)	6 (3.0%)
Pakistani	306 (8.5%)	1 (0.3%)	157 (51.3%)	106 (34.6%)	42 (13.7%)
Afghan	303 (8.4%)	0 (0.0%)	131 (43.2%)	149 (49.2%)	23 (7.6%)
Indian	297 (8.3%)	1 (0.3%)	173 (58.2%)	113 (38.0%)	10 (3.4%)
Bangladeshi	350 (9.7%)	16 (4.6%)	266 (76.0%)	65 (18.6%)	3 (0.9%)
Filipino	379 (10.5%)	1 (0.3%)	211 (55.7%)	163 (43.0%)	4 (1.1%)

* There were statistically significant differences (*p* values ˂ 0.001) in body weight status among participants from different countries.

**Table 3:** Matrix of post hoc association coefficients for different pairs of participant subgroups based on their nationality.

Study Participants by Nationality	Saudi	Egyptian	Yemeni	Syrian	Jordanian	Sudanese	Turkish	Pakistani	Afghan	Indian	Bangladeshi
**Saudi**	1.000 * 1.000 **										
**Egyptian**	**0.001** **0.001**	1.000 1.000									
**Yemeni**	**0.001** **0.028**	**0.001** **0.001**	1.000 1.000								
**Syrian**	0.434 0.871	**0.001** **0.001**	**0.002** **0.018**	1.000 1.000							
**Jordanian**	**0.001** 0.246	**0.001** **0.004**	**0.001** **0.001**	**0.001** 0.316	1.000 1.000						
**Sudanese**	**0.001** **0.001**	**0.001** 0.910	**0.001** **0.001**	**0.001** **0.001**	**0.030** **0.006**	1.000 1.000					
**Turkish**	**0.001** 0.080	**0.001** **0.001**	0.446 0.861	**0.003** 0.057	**0.009** **0.005**	**0.001** **0.001**	1.000 1.000				
**Pakistani**	0.121 0.542	**0.001** **0.001**	**0.001** 0.111	0.125 0.438	**0.001** 0.075	**0.001** **0.001**	**0.001** 0.222	1.000 1.000			
**Afghan**	**0.001** 0.150	**0.011** **0.007**	**0.001** **0.001**	**0.049** 0.200	**0.004** 0.798	**0.001** 0.011	**0.003** **0.002**	**0.001** **0.038**	1.000 1.000		
**Indian**	**0.001** **0.022**	**0.001** **0.001**	0.522 0.864	**0.001** **0.014**	**0.001** **0.001**	**0.001** **0.001**	0.830 0.748	**0.001** 0.086	**0.001** **0.001**	1.000 1.000	
**Bangladeshi**	**0.001** **0.001**	**0.001** **0.001**	**0.001** **0.001**	**0.001** **0.001**	**0.001** **0.001**	**0.001** **0.001**	**0.001** **0.001**	**0.001** **0.001**	**0.001** **0.001**	**0.001** **0.001**	1.000 1.000
**Filipino**	**0.001** 0.081	**0.001** **0.001**	**0.006** 0.595	**0.001** 0.054	**0.023** **0.003**	**0.001** **0.001**	0.300 0.780	**0.001** 0.261	**0.001** **0.001**	0.137 0.490	**0.001** **0.001**

* Association coefficients were determined using the Chi-squared test when body weight status was categorized into underweight, normal weight, overweight and obesity. Differences were considered statistically significant at *p* value < 0.05 and significant values are presented in **Bold type**. ** Association coefficients were determined using the Chi-squared test when body weight status was categorized into non-overweight/non-obese and overweight/obese. Differences were considered statistically significant at *p* value < 0.05 and significant values are presented in **Bold type.**

**Table 4:** Risk of overweight and obesity among study participants for sociodemographic variables.

Variables	Unadjusted Odds Ratio *	95% CI	*p* Value	Adjusted Odds Ratio **	95% CI	*p* Value
Participants’ Nationality						
Bangladeshi	1.00			1.00		
Saudi	4.29	3.02–6.10	˂**0.001**	3.90	2.54–5.98	˂**0.001**
Egyptian	8.60	5.99–12.35	˂**0.001**	7.37	4.98–10.90	˂**0.001**
Yemeni	3.01	2.14–4.24	˂**0.001**	2.76	1.92–3.96	˂**0.001**
Syrian	4.41	3.11–6.26	˂**0.001**	3.72	2.50–5.53	˂**0.001**
Jordanian	5.22	3.66–7.44	˂**0.001**	4.15	2.78–6.20	˂**0.001**
Sudanese	8.43	5.85–12.14	˂**0.001**	7.59	5.24–10.98	˂**0.001**
Turkish	3.11	2.12–4.57	˂**0.001**	2.43	1.63–3.61	˂**0.001**
Pakistani	3.89	2.75–5.50	˂**0.001**	3.82	2.68–5.44	˂**0.001**
Afghan	5.45	3.84–7.72	˂**0.001**	5.42	3.80–7.73	˂**0.001**
Indian	2.93	2.06–4.17	˂**0.001**	2.85	1.99–4.07	˂**0.001**
Filipino	3.27	2.34–4.56	˂**0.001**	3.22	2.21–4.68	˂**0.001**
Age (years)	1.10	1.08–1.12	˂**0.001**	1.07	1.04–1.10	˂**0.001**
Residency Duration (years)	1.02	1.01–1.03	˂**0.001**	1.05	1.03–1.08	˂**0.001**
Household Type						
Non-family household	1.00			1.00		
Family household	1.40	1.18–1.66	˂**0.001**	0.87	0.69–1.11	0.262
Marital Status						
Single	1.00			1.00		
Married	1.32	1.16–1.50	˂**0.001**	1.04	0.87–1.23	0.689
Educational Level						
Low (secondary school or less)	1.00			1.00		
High (college degree or more)	1.53	1.34–1.76	˂**0.001**	1.22	0.99–1.51	0.061
Monthly Income						
Low (˂USD 1000)	1.00			1.00		
High (≥USD 1000)	1.57	1.35–1.82	˂**0.001**	1.21	0.99–1.48	0.067

* Univariate logistic regression analysis was used to test differences between overweight/obese versus non-overweight/ non-obese (reference group). Differences were considered statistically significant at *p* value < 0.05, and significant values are presented in **Bold type**. ** Multivariate logistic regression analysis was used to test differences between overweight/obese versus non-overweight/ non-obese (reference group) after adjusting for the following sociodemographic variables: nationality, age, residency duration, household type, marital status, educational level, and monthly income. Differences were considered statistically significant at *p* value < 0.05, and significant values are presented in **Bold type.**

## Data Availability

All data generated or analyzed during this study are included in this article. If someone wants to request the data from this study can contact the corresponding author.
